# The Post-Healing Follow-Up of Diabetic Foot Ulcers by a Multidisciplinary Team to Reduce Their Recurrence: An Observational Retrospective Study

**DOI:** 10.3390/jcm14144975

**Published:** 2025-07-14

**Authors:** Marie Bouly, Francois-Xavier Laborne, Caroline Tourte, Elodie Henry, Alfred Penfornis, Dured Dardari

**Affiliations:** 1 Diabetology Department, Hopitalier Sud Francilien, 91106 Corbeil-Essonnes, France; marie.bouly@chsf.fr (M.B.); alfred.penfornis@chsf.fr (A.P.); 2 Unite’ de Recherche Clinique, Centre Hospitalier Sud Francilien, 91106 Corbeil-Essonnes, France; francois-xavier.laborne@chsf.fr (F.-X.L.); caroline.tourte@chsf.fr (C.T.);; 3 Paris-Sud Medical School, Paris-Saclay University, 91190 Gif-Sur-Yvette, France; 4 LBEPS, University Evry, IRBA, Universite’ Paris Saclay, 91100 Evry, France

**Keywords:** diabetes complications, multidisciplinary follow-up, diabetic foot disease, recurrence rate

## Abstract

**Background**: Diabetic foot disease is a public health problem. The challenges of its management lie in the complexity of wound healing and, in particular, the high rate of lesion recurrence. **Objectives**: The primary objective of the study was to evaluate whether optimized post-healing follow-up by a multidisciplinary team can reduce the recurrence rate of foot ulcers in people living with diabetes. The secondary objectives were to assess patient needs in terms of hospitalization for recurrence, the number of amputations, pedicure care, and the use of adapted footwear. **Participants**: The study included 129 patients with diabetes presenting a healed foot ulcer. A total of 38 patients underwent an annual post-healing follow-up visit with a multidisciplinary team (optimized follow-up), while 91 had a visit every 2 years (minimum follow-up). **Results**: Of the 38 patients with optimal follow-up, 8 presented a wound recurrence (21.1%) compared with 38 out of 91 patients (41.8%) receiving minimum follow-up. The recurrence rate decreased significantly between the two groups (*p* < 0.05). The use of adapted shoes was also significantly better in the group with optimized follow-up (*p* = 0.02). **Conclusions**: Regular post-healing follow-up with a multidisciplinary team seems to be a contributing factor to reducing the recurrence of diabetic foot ulcers among people living with diabetes.

## 1. Background

Diabetic foot disease is one of the major complications of diabetes with a high risk of amputation. Indeed, 85% of amputations in diabetic patients are preceded by a diabetic foot ulcer (DFU), while amputation takes place in the world every 20 s [1]. In addition, 15 to 20% of diabetic patients present a foot ulcer during their lifetime [2]. The 5-year mortality for diabetic patients with DFU is 2.5 times higher than for those without an ulcer [3]. The overall prevalence of DFU among diabetic patients is 6.3%, with a predominance in men and type 2 diabetes [4].

The management of DFU has improved over the past decades, with a recent study showing that patients with a foot lesion have a 5-year survival rate of 85% (vs. 55% before 2000) as well as a lower rate of amputation, more cases of minor amputation, and better revascularization rates [5]. The recurrence of DFU is very frequent in these patients with a high risk of foot ulceration. A recent meta-analysis reported an annual recurrence rate of 22% per person [6]. Armstrong et al. indicated that the risk of recurrence was 30 to 40% in the 1st year after healing, 50% at 2 years, 60% at 3 years, and 65% at 5 years [7]. The high rate of recurrence may be explained by the ongoing presence of the factors causing the first wound, even after healing. Indeed, as the peripheral neuropathy, foot deformations, and plantar hyperpressure have not disappeared, patients are still at high risk of diabetic foot disease, equally because of the role of atherosclerosis as a factor in the recurrence of UPD [8].

The International Working Group on the Diabetic Foot (IWGDF) suggested that 75 to 80% of cases of DFU and its recurrence could be avoided if patient follow-up had taken into account the different recommendations supported by clinical trials [9]. Various approaches have been developed to help reduce the recurrence rate with the involvement of numerous medical specialties and healthcare professionals, not to mention the patients themselves. Patient involvement seems crucial in recurrence prevention programmes, with consideration of their preferences and skills. It is also necessary to understand the reasons behind their non-adherence to the recommended care [10].

Commenting on its meta-analysis on the prevention of wounds and their recurrences in 2020 [10], the IWGDF noted that only five new studies on DFU prevention had been conducted in the past 4 years, even though its 2016 recommendations stressed the urgent need for studies on the subject [11]. Consequently, there is a real need to study post-healing follow-up to evaluate whether this type of monitoring can reduce the recurrence rate of foot lesions among patients living with diabetes.

## 2. Methods and Results

The primary objective of this study was to evaluate the impact of the optimal multidisciplinary follow-up of patients with a healed DFU on wound recurrence compared with the rate of recurrence among patients undergoing minimum follow-up by a multidisciplinary team. Our secondary goals were to evaluate patient needs in terms of hospitalization for recurrence, the number of amputations, pedicure care according to the recommendations, and the use of adapted shoes.

### 2.1. A Description of the Study Protocol

This observational retrospective study was conducted between 2016 and 2020 at the Diabetology Department of the Centre Hospitalier Sud Francilien, France. Complete information about the study was provided to each patient in a specific printed document. Since 2016, the Diabetology Department has provided a specialized consultation every 6 months to all patients in remission for DFU. This study evaluated the pertinence of this multidisciplinary follow-up with a team composed of a podiatrist, a pedorthist, an orthoprosthetist, a nurse specialized in the management of diabetic foot disease, and, if necessary, a diabetologist.

Two patient groups were defined based on the number of consultations over a 2-year period. Patients who had completed at least two follow-up sessions with the multidisciplinary team over the course of the 2 years were included in the group with an optimal follow-up (group 1, G1), while those who had had only one consultation were included in the group with a minimum follow-up (group 2, G2).

The protocol of the study was approved by the ethics committee of the Centre Hospitalier Sud Francilien, France, and was recorded in the system of protocol records under the number NCT04892771.

#### Inclusion Criteria

We included patients aged 18 years and older with type 1 or 2 diabetes followed at the Centre Hospitalier Sud-FranciIien (CHSF), France. The patients had been treated in-hospital for foot wounds between 2017 and 2019 and had received post-healing follow-up for 2 years. All included patients were informed about the study and gave consent for participation.

### 2.2. Data Collection

Data were collected from the electronic patient files from the Diabetology Department over a period of 2 years. The following data were collected: the verification of the inclusion criteria, age, sex, the type and duration of diabetes, arterial disease, diabetic neuropathy, the history of amputation, and glycosylated hemoglobin.

During the 2-year observation period from the date of inclusion, the following information was collected: the number of consultations, the recurrence of foot wounds, hospitalization for DFU, pedicure care, and the use of adapted footwear.

### 2.3. Statistical Methods

To detect a difference of 25 to 40% between the two groups with an alpha risk of 5%, a power of 80%, and correction for continuity, 158 patients were needed. We estimated that 200 diabetic patients were monitored by the multidisciplinary team at the hospital for diabetic foot remission, which would allow us to include a sufficient number of patients.

Categorial variables are presented as numbers and percentages and were compared using Fisher’s exact test or chi-squared test. Quantitative variables are presented as medians and interquartile ranges or means and standard deviations and were compared using the t-test or Wilcoxon test depending on their distribution. Recurrences, hospitalization, pedicure care, and the use of adapted shoes were compared between the two groups using Fisher’s exact test.

Time to recurrence in each group was measured using the Kaplan–Meier method and compared between the two groups using the log-rank test.

All tests were two-sided, with an alpha risk of 5%. Statistical analysis was performed using R R-4.5.1 software.

## 3. Results

### 3.1. Participants

A total of 129 participants were included in the study. The characteristics of participants are summarized in Table 1.

Overall, 38 patients received optimal follow-up over 2 years with at least an annual consultation with the multidisciplinary team (G1), whereas 91 patients underwent minimum follow-up with only one follow-up visit over the observation period (G2). The two groups presented a similar clinical profile with the presence of neuropathy in 100% of participants. Arteriopathy was comparably represented in the two groups with 16 patients in G1 (42.1%) versus 49 patients in G2 (53.8%).

### 3.2. Primary Study Objective

The results of the primary study objectives are presented in Table 2. During the 2-year follow-up period, the recurrence rate of DFU was significantly lower in G1 with 8 patients (21.1%) compared with 38 patients (41.8%) in G2 (*p* = 0.03) (Table 2). Furthermore, the time to recurrence was also significantly longer in G1 with 623 days compared with 333 days in G2 (*p* < 0.01).

### 3.3. Secondary Objectives

Regarding the secondary objectives of the study (Table 2), 8 patients (21.1%) in G1 were amputated during the 2-year follow-up period, compared with 31 (34.1%) in G2, although the difference was non-significant (*p* = 0.21). Only 1 patient (2.6%) in G1 was hospitalized for DFU recurrence compared with 14 (15.4%) in G2, with the difference being once again non-significant (*p* = 0.07). A non-significant difference was observed for pedicure care, with 28 (80%) versus 59 patients (69.4%) in G1 and G2, respectively, following the recommendations (*p* = 0.27). Finally, the use of adapted shoes showed a significant between-group difference, these being worn by 26 patients (74.3%) in G1 versus 43 (50.6%) in G2 (*p* = 0.02).

## 4. Discussion

DFU affects around 18.6 million people worldwide each year [12]. This ulceration is associated with poorer physical function, a reduced quality of life, and the greater use of healthcare services [13]. If left untreated, DFU can lead to soft tissue infection or even gangrene. Ndosi et al. previously demonstrated that a multidisciplinary team care approach (specific health services for DFU) reduced diabetes-related amputations of the lower limbs [14]. Although the composition and activities of the multidisciplinary team may vary, it generally includes at least one specialist physician in addition to a nurse, a podiatrist, and a pedorthist; this team may also include other medical professionals such as an infectiologist, a vascular physician, an orthopedist, or a vascular surgeon. The management of DFU patients by multidisciplinary teams has proven its effectiveness in reducing the amputation rate, as shown in a systematic review of 18 studies involving 38,608 participants [15], although the impact of the multidisciplinary follow-up on reducing the recurrence rate was not evaluated. In a study by Armstrong et al., the reduction in the recurrence rate was estimated at 42% at 1 year and 65% at 5 years thanks to the follow-up by a multidisciplinary team [7]. A recent longitudinal study of 129 patients in remission for DFU revealed that only 17% experienced a recurrence in the same foot compared with 48% in the contralateral foot [16].

The high rate of DFU recurrence stresses the need for continuous monitoring by both the patient and the medical team [17]. In 2023, the updated recommendations of the IWGDF raised the issue of recurrence as a real challenge and a barrier in the management of DFU. The working group suggested that clinical trials should evaluate post-healing follow-up and its impact on the reduction in DFU recurrence [18].

The primary objective of our study was to evaluate the impact of post-healing follow-up on the rate of DFU recurrence. We initially proposed this follow-up with a multidisciplinary team with a consultation every 6 months, but due to a lack of participants, the follow-up was considered optimal if patients had an annual follow-up visit (G1). Patients who underwent a single consultation during the two-year follow-up period comprised the minimum follow-up group (G2). We initially discussed the idea of constituting a conventional group of participants without any post-healing follow-up. However, because the post-healing follow-up consultation is proposed to all patients after DFU healing, only the number of consultations over a 2-year period was taken as the evaluation criteria for the primary study objective. The two groups had a similar demographic and clinical profile in terms of the neuropathy and obliterating arteriopathy of the lower members.

Our results demonstrated a significant decrease in the recurrence rate of DFU as well as the time to recurrence when patients benefited from post-healing consultations at least annually compared with the follow-up performed only once over the 2-year follow-up period. The rates of recurrence at 1 and 2 years [7] were known without taking into account post-healing follow-up of the first presentation of UPD. The findings also showed the better use of adapted shoes in patients receiving the optimal post-healing follow-up compared with those in the minimum follow-up group.

Our observational study at least partially showed that the multiplication of post-healing follow-up consultations can contribute to decreasing the recurrence rate of DFU. Our study may also open up discussions about the advantages of this type of multidisciplinary follow-up from an economic perspective, with the team being composed of a single specialist physician (i.e., the referring diabetologist) along with paramedical specialists.

This follow-up mode represents a glimmer of hope for reducing the number of cases of people living with diabetes complicated by UPC. In France, for example, there has been a sharp increase in hospital admissions related to diabetic foot disease, with a significant increase in healthcare costs for its treatment.

Regarding the limitations of this study, its retrospective nature and the absence of randomization between the two groups may be considered limitations, and it is also difficult to determine the contributing factors leading to this significant reduction in wound recurrence in the group with optimal follow-up, such as patient compliance, the wearing of appropriate footwear, etc. The exclusion of the entire population initially included in the statistical power of the study (129/158) probably impacted the comparison of the two groups regarding the secondary characteristics of the study.

## 5. Conclusions

Despite its retrospective nature and the influence of other factors that may influence assessment, this study demonstrated that regular post-healing follow-up by a multidisciplinary team seems to be a contributing factor in reducing the recurrence rate of DFU in people living with diabetes, as well as the time to recurrence.

## Figures and Tables

**Table 1:** The characteristics of the patients included in the study.

Sex	Overall Population	Optimal Follow-Up	Minimum Follow-Up
	N = 129	N = 38	N = 91
Women	32 (24.8)	9 (23.7)	23 (25.3)
Men	97 (75.2)	29 (76.3)	68 (74.7)
Mean age (standard deviation)	66.74 (11.38)	68.27 (10.92)	66.11 (11.57)
Diabetes			
Type 1	9 (7)	4 (10.5)	5 (5.5)
Type 2	120 (93)	34 (89.5))	86 (94.5)
Median duration of diabetes [IQ] (N = 111)	19 [12.5–22.5]	18 [13–27]	19 [12–22]
Neuropathy	129 (100)	38 (100)	91 (100)
Arteriopathy	65 (50.4)	16 (42.1)	49 (53.8)
Amputation	39 (30.2)	8 (21.1)	31 (34.1)
Hospitalization	15 (11.6)	1 (2.6)	14 (15.4)
Median days of hospitalization [IQ] (N = 15)	15 [5.5–17]	15 [15–15]	14.55 [5.25–17]
Median HbA1C [IQ] (N = 110)	7.5 [6.8–8.5]	7.2 [6.28–8.63]	7.6 [7.0–8.5]

**Table 2:** The results for the two groups with the optimal versus minimum follow-up.

Variables	Optimal Follow-Up	Minimum Follow-Up	*p*-Value	OR	IC95%
	N = 38	N = 91			
Diabetic foot ulcer recurrence	8 (21.1)	38 (41.8)	0.03	0.32	[0.11–0.84]
Median time to recurrence (days) [IQ] (N = 46)	623 [440–643.5]	333.0 [240.2–455.5]	<0.01	0.38	[0.15–0.89]
Pedicure care (N = 120)	28 (80)	59 (69.4)	0.06	1.88	[0.71–5.2]
Adapted shoes	26 (74.3)	43 (50.6)	*p* = 0.02	0.61	[0.21–1.71]
Death	1 (2.6)	3 (3.3)	0.57		

## Data Availability

All data is contained within the article.
